# Ineffectiveness of Intermittent Hemodialysis in a Critically Ill COVID-19 Patient: A Case of Persistent Heparin-Induced Hyperkalemia

**DOI:** 10.1155/2022/8613656

**Published:** 2022-03-04

**Authors:** Yannick M. Nlandu, Yannick M. Engole, Marie-France I. Mboliassa, Théodore-Junior M. Sakaji, Patrick U. Kobo, Patrick M. Boloko, Pally K. Mafuta, Joseph P. Tsangu, Karel Van Echkout, Jean-Pierre M. Kanku, Golan Kalifa, Rodolphe Ahmed, Justine B. Bukabau

**Affiliations:** ^1^Intensive Care Unit, Centre Médical de Kinshasa, Kinshasa, Democratic Republic of the Congo; ^2^Nephrology Unit, Kinshasa University Hospital, Kinshasa, Democratic Republic of the Congo; ^3^Intensive Care Unit, Kinshasa University Hospital, Kinshasa, Democratic Republic of the Congo

## Abstract

Heparin is widely used in the intensive care unit despite the risk of bleeding it can cause. Although it is rarely reported, hyperkalemia is one of the side effects associated with heparin therapy (unfractionated or fractionated heparin). It would be secondary to hypoaldosteronism by blocking the biosynthesis of aldosterone in the adrenal gland and often appears in context of prolonged heparin therapy or inappropriate renin production in elderly, diabetic, and kidney insufficiency patients. We report a case of persistent hyperkalemia in a diabetic COVID-19 patient treated with curative heparin in the context of severe COVID-19.

## 1. Introduction

Hyperkalemia is a potentially life-threatening electrolyte disorder [1]. Factors associated with its development include altered renal clearance of potassium and release from the intracellular space, as well as altered transfer to the intracellular space. [1]. During acute kidney injury (AKI) and particularly in hemodialysis (HD), hyperkalemia can be persistent in some situation such as rhabdomyolysis or tumor lysis syndrome [1]. We report the case of a refractory hyperkalemia to intermittent hemodialysis (IHD) in a COVID-19 patient without any situation of continuous potassium release.

## 2. Case Presentation

A 72-year-old European man presented to the Centre Médical de Kinshasa (CMK) for progressive respiratory distress since June 29, 2021, associated with generalized weakness. His past medical history was remarkable for diabetes mellitus type II, hypertension, and unspecified arrhythmia for the past ten years. His medications included the oral antidiabetic drug repaglinide and the antihypertensive drugs ibesartan, hydrochlorothiazide, manidipine, and flecainide. A nasopharyngeal swab sample was positive for COVID-19 mRNA by reverse transcriptase-PCR (RT-PCR), and the patient was initially managed at home with azithromycin, rivaroxaban, vitamin C, vitamin D, prednisone, and oxygen for six days. The course was marked by worsening dyspnea, and the patient visited CMK on July 7, 2021. On hospital admission, he was dyspneic with a respiratory rate of 33 breaths per minute. His blood pressure was 145/74 mmHg, and peripheral oxygen saturation was 75% despite the 15 L/min of oxygen. Pulmonary auscultation was marked by crackling rales, and the patient also had lower limb edema. The blood sample revealed elevated cardiac and inflammatory biomarkers and respiratory alkalosis with hypoxemia. The chest CT scan argues for a very significant COVID-19 pneumonia stage 5 without pulmonary embolism. There was no kidney dysfunction, significant proteinuria, or hyperkalemia. Initial laboratory assessment and selected trends are depicted in Table 1.

A diagnosis of severe COVID-19 was made, and the patient was managed with a noninvasive ventilation with high level of oxygen, dexamethasone, vitamin C, vitamin D, empirical antibiotic (ceftriaxone plus ofloxacin was used as the first line of antibiotic, and imipenem, amikacin, and vancomycin were used as the second line), and curative anticoagulation dose (1 mg/kg) of enoxaparin subcutaneously every 12 h started at admission. Anti-Xa activity of enoxaparin was not available. Ten days after his admission, he experienced a respiratory degradation with severe hypoxemia, shock, and acute kidney injury (AKI) which required, respectively, intubation with mechanic ventilation and vasoactive drugs (norepinephrine and dobutamine). On July 21, the patient started hemodialysis consecutively to the worsening of AKI with progressive anuria associated with hypervolemia and hyperkalemia refractory to medical strategy (insulin-dextrose, *β*2 agonist, and sodium polystyrene sulfonate). Ibesartan was stopped for suspected drug-induced hyperkalemia. Even during hemodialysis (was made every day) combined to medical strategy, hyperkalemia remained persistent in the absence of rhabdomyolysis, tumoral lysis syndrome, or metabolic acidosis and was associated to hyponatremia. The patient had a total of 7 sessions (one by day) of combined hemodiafiltration (4) to classic hemodialysis (3), 3 to 4 hours per day with a plasma and dialysate flow rates in order of 200–250 and 400–500 mL/min, respectively. The daily KT/V by online clearance measurement varied between 0.6 and 0.7. The dialyzer used was made of polysulfone or high-flux membrane, and the dialysate had a lower potassium concentration of 2 mmol/L and a high bicarbonate concentration of 36 mmol/L. Unfractionated heparins were used for anticoagulation during the hemodialysis session. On July 28, the patient was transferred abroad. One day after, he died in context of a severe hypoxemia.

## 3. Discussion

SARS-CoV-2 infection is currently recognized to be a multivisceral disease, which can affect several organs due to the wide distribution of angiotensin-converting enzyme 2 (ACE2) receptors, the entry point of the virus into the human cell [2]. In the kidney, the SARS-CoV-2 virus can be responsible for various lesions, including the proximal tubulopathy responsible for hypokalemia [3]. In the AKI context, hyperkalemia is the most expected complication. However, during dialysis, its incidence is reduced and gradually decreased with the kidney function recovery. The persistence of hyperkalemia during dialysis is most often associated with a situation of continuous release of potassium from the intracellular to the extracellular space such as in rhabdomyolysis or tumor lysis syndrome [1]. The improvement of kidney replacement therapy (KRT) is favorable to better potassium clearance. Indeed, the potassium dialysance depends not only on its gradient between the plasma and the dialysate, on their respective flow rates, or on the characteristics of the dialyzer but also on the different dialysis modalities (hemodialysis, hemofiltration, or hemodiafiltration) [1]. The potassium mass transfer depends on the time of dialysis as well as on the intracellular potassium kinetics described according to a multicompartimental model, explaining the phenomenon of rebound in postdialysis significantly reduced by continuous dialysis techniques [1]. The use of intermittent dialysis, therefore, requires, on one hand, high plasma and dialysate flow rates and, on the other hand, the extension of its duration in the context of uncontrolled hyperkalemia cause such as rhabdomyolysis and tumor lysis syndrome [1]. The persistence of hyperkalemia in a COVID-19 patient was also reported by Siddharth et al. and could be explained by the use of IHD [4]. However, even for a prolonged sustained low-efficiency dialysis (SLED), Ramanand et al. reported a higher incidence of hyperkalemia in the COVID-19-AKI group compared to the AKI group of other causes [5]. According to them, refractory hyperkalemia was secondary to a continuous release of intracellular ions from the damaged tissues associated with the COVID-19 cytokine storm as shown by the correlation between potassium, phosphorus, and elevated lactate dehydrogenase (LDH) levels reported in their cohort as well as the use of a noncontinuous dialysis technique. The reverse kinetic in our patient of LDH with normal creatine kinase (CK) and uric acid level compared to the occurrence of a concomitant episode of hyperkalemia at the beginning of dialysis suggests another hypothesis than tissue damage. Acidemia causes an extracellular shift of potassium and can, thus, explain hyperkalemia in our patient. However, respiratory acidosis rarely causes clinically significant hyperkalemia [6]. Indeed, during respiratory acidosis, rapid cell entry of CO_2_ will acidify intracellular pH that, by stimulating Na^+^ entry by Na^+^-H^+^ exchange, tends to enhance Na^+^/K^+^-ATPase activity and oppose a net loss of intracellular K^+^. Heparin therapy may be the cause of this hyperkalemia. Indeed, the prothrombotic profile of the severe COVID-19 patient associated with a significant incidence of thromboembolic events justifies the use of curative anticoagulation [7]. Hyperkalemia secondary to heparin is a rarely reported side effect. It is secondary to a state of hypoaldosteronism following a reduction in the number and the affinity of angiotensin II receptors in the glomerular zone of the adrenals and, therefore, responsible for an increase in kidney retention of potassium [8]. This process is strongly pronounced in the elderly, diabetic patients, and patients with kidney failure who were unable to adequately increase renin production [9]. The risk is even greater for large and prolonged doses of heparin [7]. The pathophysiological mechanisms of hyponatremia among patients with COVID-19 are diverse, including syndrome of inappropriate antidiuretic hormone secretion (SIADH), digestive loss of sodium ions, reduced sodium ion intake, or use of diuretic therapy. Hyponatremia in our context can be associated to the hypoaldosteronism state. The trend of acid-base alterations in our COVID-19 patient with pneumonia is in line to lung disease that cause abnormalities in alveolar gas exchange without an alveolar hypoventilation and are characterized by a ventilation stimulation due to hypoxia responsible of a respiratory alkalosis. Hypercapnia typically occurs late in the disease process with severe pulmonary disease or when respiratory muscle fatigue occurs. The lack of the metabolic acidosis component in the context of an anuric patient is exceptional but can be explained in our context by the daily dialysis with an optimal dialysate bicarbonate concentration.

## 4. Conclusions

Hyperkalemia is associated with morbidity and mortality and requires identifying possible causes in order to provide appropriate care to patients. We report a case of heparin-induced hyperkalemia in critically ill COVID-19 patients.

## Figures and Tables

**Table 1:** Summary of laboratory evaluations and relevant trend during hospitalization.

Date	Reference	July 7, 2021	July 9, 2021	July 10, 2021	July 12, 2021	July 14, 2021	July 17, 2021^*∗*^	July18, 2021	July 19, 2021	July 20,2021^*∗∗*^	July 21,2021	July 22, 2021	July 23, 2021	July 24, 2021	July 25, 2021	July 26,2021
Arterial pH	7.35–7.45	7.62	7.45	7.50	7.50	7.45	7.5	7.41	7.44	7.29	7.33	7.36	7.37	7.38	7.35	7.16
pCO2 (mmHg)	35–45	27.4	47.8	49.7	43.3	43	27	44.7	52.9	61.3	53	48	83	84	88	63
HCO3 (mmol/L)	22–32	27.4	33.7	39.4	33.8	30.6	27	28	32.8	24.9	25	28	28	26	26	25.8
Hemoglobin (g/dl)	12.5–15			15.9		16.3	14.8	14.4		14.3		13.6				12.0
White blood cell count (elements/mm^3^)	4,000–10,000	13870		13080		14940	24370	18100		18.500		25.570				27.150
Glycated hemoglobin (%)	4–5.6	7.1														
Fibrinogen (g/L)	2–4	5.5														
D-dimer (ng/mL)	220–500	˃10000			1224.2											
Procalcitonin (ng/mL)	˂0.15	0.18		0.25	0.17	0.10	0.28			0.74			0.41			0.98
CRP (mg/L)	˂6	147		66	41	23	105			297			71			132
Blood urea (mmol/L)	2.5–7.5	10.6			14	14.3	18.6	19.5	14.9	19.8	26.4		25.8			20.3
Serum creatinine (*μ*mol/L)	53–120	69			95	75	126	123	94	170	292		336			290
Sodium (mmol/L)	135–145	129	139	138	139	131	129	134		134	136	134	135	135	134	137
Potassium (mmol/L)	3.0–5.0	4	4.5	4.8	4.7	4.8	4.2	4.5		5.1	6.6	6.4	6.0	6.0	6.4	5.9
Ionized calcium (mmol/L)	1.15–1.30	1.09			1.20											
Aspartate amino transferase (UI/L)	6–34	32									45					
Alanin amino transferase (UI/L)	8–38	29									41					
Uric acid (*μ*mol/L)	150–420	320									332					
Phosphorus (mmol/L)	0.97–1.45	1.10									1.7					
LDH (UI/L)	140–280	936				674	450				441					
CK (UI/L)	22–198	101					97				82					
Troponin (Abbot) (ng/mL)	0–0.4	258.3	138.2													
Brain natriuretic peptide (pg/mL)	˂300	2071	770			2179	4334	3232	1040	1027	2169					
PCR-COVID-19		+				—										—
Antigen/IgG COVID-19		—														
Antigen/IgM COVID-19		+														

^*∗*^Day of intubation. ^*∗∗*^Start of hemodialysis.

## Data Availability

The data supporting the findings of the study are available within the article and can be obtained by emailing the corresponding author.
